# Differential impact of prenatal PTSD symptoms and preconception trauma exposure on placental *NR3C1* and *FKBP5* methylation

**DOI:** 10.1080/10253890.2024.2321595

**Published:** 2024-04-26

**Authors:** Laura R. Stroud, Nancy C. Jao, L. G. Ward, Sharon Y. Lee, Carmen J. Marsit

**Affiliations:** a COBRE Center for Stress, Trauma, and Resilience*, Center for Behavioral and Preventive Medicine, The Miriam Hospital, Providence, RI, USA; b Department of Psychiatry and Human Behavior, Alpert Medical School, Brown University, Providence, RI, USA; c Department of Psychology, Rosalind Franklin University of Medicine and Science, North Chicago, IL, USA; d Gangarosa Department of Environmental Health, Emory University Rollins School of Public Health, Atlanta, GA, USA

**Keywords:** *NR3C1*, *FKBP5*, placenta, trauma, PTSD, pregnancy

## Abstract

Perinatal stress is associated with altered placental methylation, which plays a critical role in fetal development and infant outcomes. This proof-of-concept pilot study investigated the impact of lifetime trauma exposure and perinatal PTSD symptoms on epigenetic regulation of placenta glucocorticoid signaling genes (*NR3C1* and *FKBP5).* Lifetime trauma exposure and PTSD symptoms during pregnancy were assessed in a racially/ethnically diverse sample of pregnant women (*N* = 198). Participants were categorized into three groups: (1) No Trauma (−T); (2) Trauma, No Symptoms (T − S); and (3) Trauma and Symptoms (T + S). Placental tissue was analyzed via bisulfite pyrosequencing for degree of methylation at the *NR3C1* promoter and *FKBP5* regulatory regions. Analyses of covariance were used to test group differences in percentages of *NR3C1* and *FKBP5* methylation overall and at each CpG site. We found a significant impact of PTSD symptoms on placental *NR3C1* methylation. Compared to the −T group, the T + S group had greater *NR3C1* methylation overall and at CpG6, CpG8, CpG9, and CpG13, but lower methylation at CpG5. The T + S group had significantly higher N*R3C1* methylation overall and at CpG8 compared to the T − S group. There were no differences between the T − S group and − T group. Additionally, no group differences emerged for *FKBP5* methylation. Pregnant trauma survivors with PTSD symptoms exhibited differential patterns of placental *NR3C1* methylation compared to trauma survivors without PTSD symptoms and pregnant women unexposed to trauma. Results highlight the critical importance of interventions to address the mental health of pregnant trauma survivors.

## 1. Introduction

It is estimated that one in every two women will experience at least one traumatic event in their lifetime (National Center for Posttraumatic Stress Disorder, 2021). Overall, women are more likely than men to experience the kinds of traumatic exposures most strongly associated with posttraumatic stress disorder (PTSD), such as sexual violence and childhood sexual abuse (Liu et al., 2017). In pregnant women, rates of perinatal PTSD are estimated to be 3.3% in community samples (Cook et al., 2018) and 18.95% in high-risk samples (Yildiz et al., 2017), with estimates as high as 27–58% for the presence of any PTSD symptoms among pregnant women with lifetime trauma exposure (Harris-Britt et al., 2004; Seng et al., 2010). Previous studies have linked both lifetime trauma exposure and perinatal PTSD symptoms to adverse maternal health and child development outcomes (Cook et al., 2018; Racine et al., 2018). In particular, perinatal PTSD symptoms may confer unique risk – above and beyond the effects of mere exposure to trauma – for the transmission of trauma-related effects.

The hypothalamic-pituitary-adrenal (HPA) axis, which regulates the neuroendocrine response to stress, plays a potentially critical role in the intergenerational transmission of trauma (Bowers & Yehuda, 2016). Glucocorticoids act as the primary hormonal modulator of the endocrine stress response by allowing, stimulating, and suppressing a stress response as well as preparing the body for future stressors (Sapolsky et al., 2000). Additionally, glucocorticoids modulate psychological responses to stress, including the encoding of stress memory (Kaouane et al., 2012) and development of PTSD (Szeszko et al., 2018). The *NR3C1* promoter is one of the main genes involved in glucocorticoid signaling and modulating the stress response and can be altered via epigenetic programming (Palma-Gudiel, Cordova-Palomera, Leza, et al., 2015). Epigenetic regulation represents the control of gene expression in a mitotically heritable fashion. The most studied form of epigenetic regulation in humans is DNA methylation, a modification of the cytosine nucleotide in a cytosine-guanine (CpG) dinucleotide sequence. DNA methylation in the promoter regions of genes is often indicative of gene silencing while DNA methylation in other genic regions can have more complicated effects on gene expression. While the majority of cellular DNA methylation is erased and then reset in a cell and tissue-specific fashion, there is evidence that some cases of de novo methylation established in one generation may be propagated into the subsequent generations or may be established in offspring due to experiences of the parents (Perez & Lehner, 2019). Lifetime trauma exposure and PTSD symptoms can lead to epigenetic changes in the regulation of the HPA axis as well as the *NR3C1* promotor gene (González Ramírez et al., 2020; Palma-Gudiel, Cordova-Palomera, Leza, et al., 2015; Sheerin et al., 2020).

Prior research has demonstrated that epigenetic mechanisms may mediate the effects of prenatal and early life stress on offspring development by promoting changes in methylation of the promoter region of *NR3C1* (Ostlund et al., 2016; Parade et al., 2021; Tyrka et al., 2012). For instance, among individuals with prenatal PTSD, infants’ cortisol levels have been associated with their *NR3C1* methylation levels (Fransquet et al., 2022). This research mirrors rodent models in which early life stress (modeled by very low levels of postnatal care) alters hippocampal glucocorticoid regulation. Specifically, early life stress increases methylation of the rodent analog of *NR3C1*, the hippocampal glucocorticoid regulation gene (exon 1_7_ of the *NR3C1* promoter), which alters binding of the transcription factor NGFI-A as well as other aspects of the stress response (Meaney et al., 2007; Szyf et al., 2007) – leading to exacerbated stress responses in offspring well into adulthood. The majority of human-based research on epigenetic programming related to maternal adversity and mental health has measured *NR3C1* promoter methylation in buccal cells, or peripheral or cord blood of infant offspring (Dereix et al., 2021; Mulligan et al., 2012; Ostlund et al., 2016; Parade et al., 2021; Tyrka et al., 2012).

The placenta offers a unique window into the maternal-fetal interface as it serves an important regulatory role in gestational development, endocrine, and metabolic transmission between mother and fetus as well as provides fetal protection from potentially harmful elements of the maternal environment (Bonnin & Levitt, 2011; Maccani & Marsit, 2009). Prenatal exposure to traumatic events, such as a natural disaster, has been associated with altered placental gene expression (Zhang et al., 2020) and reprogramming of the placental transcriptome which has been associated with child HPA dysfunction (Nomura et al., 2021). Other psychological risk factors, such as trait and state prenatal anxiety have been associated with increased methylation of placental *NR3C1* (Capron et al., 2018; Dereix et al., 2021). Associations between methylation of placental *NR3C1* and alterations in newborn neurobehavior and cortisol stress reactivity have also been demonstrated (Appleton et al., 2015; Bromer et al., 2013; Conradt et al., 2015), indicating that placental *NR3C1* methylation may mediate the effects of maternal stress on infant outcomes. However, little is known about the degree to which experiences of current PTSD symptoms during pregnancy may affect the association between maternal trauma exposure and methylation of placental *NR3C1*.

Moreover, current PTSD symptoms during pregnancy may have additional effects on methylation of placental *NR3C1* that are above and beyond the effects of trauma exposure. Some types of trauma exposure, such as early life stress, have been shown to not only strongly associate with DNA methylation, but also interact with specific genes implicated in many psychiatric disorders to predict PTSD (Caspi et al., 2010; Heim & Binder, 2012). Indeed, a high number of glucocorticoid receptors is a pre-trauma risk factor for PTSD development (van Zuiden et al., 2013; Yehuda, 2009). Prior studies have looked at the effects of either trauma exposure or PTSD on *NR3C1* methylation, but with inconsistent findings across studies of the associated CpG sites within *NR3C1* (Watkeys et al., 2018), suggesting distinct contributions of trauma exposure and trauma-related sequalae to *NR3C1* methylation. Several studies have examined the associations of maternal trauma and stress with placental *NR3C1* methylation (see Palma-Gudiel et al., 2018 for review), with some studies of specifically maternal trauma exposure reporting increased methylation (Mulligan et al., 2012; Nomura et al., 2021) and others reporting reduced methylation (Kertes et al., 2016). However, no studies to our knowledge have compared the distinct contributions of lifetime trauma exposure and PTSD symptoms to placental *NR3C1* methylation.

In addition to *NR3C1*, FK506 binding protein 5, encoded by the gene *FKBP5*, is a modulator of *NR3C1* activity and the HPA stress response, further justifying the importance of focusing on both *NR3C1* and *FKBP5* methylation in relation to lifetime trauma exposure and PTSD symptoms. When *FKBP5* binds to the glucocorticoid receptor, it decreases the ability of the glucocorticoid receptor to bind to cortisol, resulting in reduced glucocorticoid receptor sensitivity and impaired negative feedback regulation of the HPA axis. *FKBP5* demethylation has been proposed as a mediator of the combined effects of gene x environment on risk of PTSD development (Klengel et al., 2013). *FKBP5* methylation has been implicated in the intergenerational transmission of trauma exposure, with higher methylation found in Holocaust survivors compared to controls but lower methylation found in survivors’ offspring compared to the offspring of controls (Bierer et al., 2020; Yehuda et al., 2016). In placental tissue specifically, higher methylation at CpG1 and CpG2 sites in *FKBP5* intron 7 has been associated with high arousal in infants, reflecting hyperreactivity to environmental stimuli (Paquette et al., 2014).

This study is a proof-of-concept pilot study investigating the differential impact of trauma exposure in addition to current PTSD symptoms during pregnancy vs trauma exposure without current PTSD symptoms on methylation of the promoter region of placental *NR3C1*. We hypothesize that trauma exposure combined with current PTSD symptoms will lead to greater methylation of placental *NR3C1*, indicating potential changes in methylation for the fetus, compared to trauma exposure without PTSD symptoms. Additionally, given the role of *FKBP5* in altering the ­glucocorticoid receptor’s responsiveness to stress signaling, we conducted exploratory analyses to test for differential impact of trauma exposure and current PTSD symptoms *vs.* trauma exposure without PTSD symptoms on placental *FKBP5* methylation.

## 2. Methods

### 2.1. Participants

Participants were English-speaking, primarily low-income pregnant women from a racially and ethnically diverse sample who completed the epigenetic sub-study of a prospective study examining the effect of maternal behaviors in pregnancy and infant behavioral development over the first postnatal month. Participants were recruited from obstetrical offices, health centers, and community postings across southern New England. Pregnant women were eligible for enrollment if they were between 18 and 40 years old, had a singleton pregnancy, did not have current/prior involvement with child protective services, and had no illicit drug use besides marijuana or serious medical conditions (e.g. pre-eclampsia and severe obesity). Participants provided written informed consent, and followed procedures reviewed and approved by Institutional Review Boards at Women and Infants Hospital and Lifespan Hospitals of Rhode Island. See Supplementary Table 1 for enrollment flow chart.

### 2.2. Study procedures and measures

#### 2.2.1. Maternal and infant characteristics

Pregnant women were interviewed prospectively over second and third trimesters of pregnancy and at delivery (*M* = 3 interviews (range 2–4) between 24 and 42 weeks gestation). At each interview, participants completed the calendar/anchor-based Timeline Follow Back (TLFB) interview regarding smoking, drug, and alcohol use over pregnancy and three months prior to conception (Robinson et al., 2014). Participants also completed a socioeconomic status (SES) interview from which education, occupation, income, and Hollingshead four-factor index of SES were extracted (Gottfried, 1985). Information regarding maternal and infant health and medical conditions (e.g. pre-eclampsia and gestational diabetes) was extracted by medical chart review. Tobacco use during pregnancy was confirmed using cotinine from maternal saliva collected at each interview as well as post-birth infant meconium.

#### 2.2.2. Self-reported trauma history and symptoms

Utilizing the Psychiatric Diagnostic Screening Questionnaire (PDSQ) self-report measure (Zimmerman & Mattia, 2001), participants were asked whether they have ever experienced or witnessed a potentially traumatic event (e.g. combat, rape, assault, sexual abuse). Participants who endorsed experiencing or witnessing a potentially traumatic event were asked whether they experienced any of the 13 symptoms based on Diagnostic and Statistical Manual of Mental Disorders IV (DSM-IV) criteria for PTSD within the last two weeks (e.g. intrusive thoughts, negative emotions, upsetting dreams, jumpiness, emotional numbness, and flashbacks related to the traumatic event) (American Psychiatric Association, 1994). Participants were then categorized into one of three trauma groups: (1) individuals who denied ever experiencing or witnessing a traumatic event were categorized in the “–T” (i.e. no trauma) group; (2) individuals who endorsed ever experiencing or witnessing a traumatic event, but denied any PTSD-related symptoms were categorized in the “T–S” (i.e. trauma without symptoms) group; and (3) individuals who endorsed ever experiencing or witnessing a traumatic event, and also endorsed experiencing any PTSD-related symptoms in the last two weeks were categorized in the “T + S” (i.e. trauma with symptoms) group.

#### 2.2.3. Placental NR3C1 and FKBP5 methylation

Following birth, placental tissue that was free from maternal decidua was excised and placed immediately in RNAlater solution at 4 degrees Celsius (Life Technologies, Grand Island, NY). After 72 h, placental tissue samples were removed from the RNAlater solution, blotted dried, snap-frozen in liquid nitrogen, pulverized to homogeneity, and stored at −80 °C. Placental genomic DNA was extracted using the QIAmp DNA Mini kit (Qiagen, Inc., Valencia, CA) and assessed for quantity and quality using a ND-1000 Spectophotometer (Nanodrop, Wilmington, DE). DNA samples were sodium bisulfite modified using the EZ DNA Methylation Kit (Zymo Research, Irvine, CA), with degree of methylation at the *NR3C1* promoter and *FKBP5* regulatory regions examined via quantitative pyrosequencing (Dupont, 2004; Oberlander, 2008) using the PyroMark MD Pyrosequencing System. The *NR3C1* promoter region analyzed encompasses exon 1 F (human homologue of rat exon 1_7_), and contains 13 CpG sites, while the regulatory region in intron 7 of *FKBP5* containing 2 CpG sites (CpG1 and CpG2) was analyzed. Methylation in both of these regions has been shown to negatively correlate with gene expression (Bromer et al., 2013; Paquette et al., 2014). See Supplementary Table 2 for corresponding genomic coordinates. Reactions were performed in triplicate, with SDs for individual sites calculated. Any sample with *SD* > 3% was re-analyzed. Sodium bisulfite-modified, fully-methylated referent positive control, and fully unmethylated (whole genome amplified) negative control DNA (Qiagen, Valencia, CA) were also included with each batch.

### 2.3. Statistical analysis

All statistical analyses were conducted using IBM SPSS Statistical version 27 (IBM Corp., Armonk, NY). Differences between the three trauma group categories (−T *vs.* T − S *vs.* T + S) in maternal and infant characteristics were assessed using chi-square and analysis of variance (ANOVA). Due to non-normal distributions, CpG methylation outcomes for *NR3C1* and *FKBP5* were natural logarithm transformed. We then compared differences in methylation percentages between trauma groups overall and at each CpG site utilizing separate analyses of covariances (ANCOVA) controlling for maternal education, gravida, and tobacco use. Post-hoc pairwise comparisons adjusted with Bonferroni corrections were also conducted to examine specific between-group differences.

## 3. Results

### 3.1. Participant characteristics

The sample included 198 pregnant women (*M*_age_ = 25.4, *SD* = 5.0) of whom 50% were tobacco users. A total of 133 (67.2%) participants denied any history of trauma or current PTSD symptoms (−T group), 23 (11.6%) participants endorsed a history of trauma but denied any current PTSD symptoms (T − S group), and 42 (21.2%) participants endorsed both a history of trauma and current PTSD symptoms (T + S group). In the T + S group, participants endorsed an average of 4.02 (*SD* = 3.21) PTSD symptoms. Participant sociodemographic characteristics overall and by group (−T *vs.* T − S *vs.* T + S) are detailed in Table 1. Regarding maternal characteristics, there were statistically significant differences in gravida (*p* = 0.006) and parity (*p* = 0.015) between trauma groups, where the T + S group reported higher gravida and parity compared to the − T group. There was also a difference in amount of tobacco use during pregnancy between trauma groups (*p* = 0.004), where 69.0% of the T + S and 60.9% of the T − S groups had tobacco use during pregnancy compared to 41.4% of the −T group. There were no significant differences between groups for any other maternal or infant characteristics (*p*s > 0.068).

**Table 1.** Participant characteristics.

	Overall (*N* = 198)	Trauma groups			*p*
		No trauma (−T; *n* = 133)	Trauma without PTSD symptoms (T − S; *n* = 23)	Trauma with PTSD symptoms (T + S; *n* = 42)	
Maternal characteristics					
Age (mean, *SD*)	25.4 (5.0)	25.3 (5.1)	26.4 (4.8)	25.4 (4.9)	0.602
Ethnicity (% Hispanic)	39.3%	37.4%	26.1%	52.4%	0.087
Race (% non-White)	60.6%	63.2%	39.1%	64.3%	0.080
% American Indian/Alaskan Native	3.0	1.5	13.0	2.4	–
% Asian	3.0	2.3	4.3	4.8	–
% Black/African American	19.7	24.1	4.3	14.3	–
% White	39.4	36.8	60.9	35.7	–
% More than one race	8.1	7.5	8.7	9.5	–
% Unknown/not reported	26.8	27.8	8.7	33.3	–
Education (% high school or less)	53%	55.6%	30.4%	57.1%	0.068
Hollingshead SES index (% low SES)	57.2%	59.2%	45.5%	57.1%	0.482
Gravida (mean, *SD*)	2.5 (1.6)	2.3 (1.5)	2.6 (1.7)	3.2 (1.8)	0.006*
Parity (mean, *SD*)	0.90 (1.09)	0.78 (.99)	0.83 (.89)	1.33 (1.39)	0.015*
Infant characteristics					
Sex (% female)	49.5 %	50.4%	36.4%	53.7%	0.398
GA in weeks (mean, *SD*)	39.4 (1.4)	39.4 (1.4)	39.6 (.85)	39.3 (1.6)	0.685
Birth weight in grams (mean, *SD*)	3351.56 (494.8)	3342.2 (490.3)	3362.6 (393.2)	3374.9 (565.2)	0.931
Small for gestational age (% yes)	5.9%	4.0%	4.5%	12.5%	0.133
Any tobacco exposure (% exposed)	49.5%	41.4%	60.9%	69.0%	0.004*

*Note.* *p < 0.05, significant differences between trauma groups based on chi-square and ANOVA analyses.

**Table 2.** Estimated means for differences between trauma groups: raw and adjusted model comparisons.

	Raw models				Adjusted models			
	Means			*p*	Means			*p*
	No trauma (−T)	Trauma without PTSD symptoms (T − S)	Trauma with PTSD symptoms (T + S)		No trauma (−T)	Trauma without PTSD symptoms (T − S)	Trauma with PTSD symptoms (T + S)	
NR3C1								
Overall	1.50	1.43	2.04	0.003*	0.37	0.35	0.40	0.009*
CpG1	0.37	0.35	0.50	0.450	0.33	0.46	0.34	0.652
CpG2	0.33	0.46	0.30	0.576	0.78	0.64	0.81	0.809
CpG3	0.78	0.64	0.99	0.379	0.19	0.35	0.21	0.753
CpG4	0.19	0.35	0.19	0.256	0.68	0.17	0.16	0.108
CpG5	0.68	0.17	0.16	0.012*	0.63	0.71	0.76	0.003*
CpG6	0.63	0.71	1.21	0.003*	3.48	3.64	3.60	0.004*
CpG7	3.48	3.64	3.96	0.103	1.40	1.31	1.56	0.268
CpG8	1.40	1.31	2.20	<0.001*	2.85	2.58	3.12	0.011*
CpG9	2.85	2.58	4.30	0.002*	2.47	2.35	2.61	0.012*
CpG10	2.47	2.35	3.21	0.010*	1.66	1.70	1.88	0.088
CpG11	1.66	1.70	2.68	0.005*	2.86	2.53	3.00	0.057
CpG12	2.86	2.53	3.71	0.014*	1.85	1.81	2.11	0.080
CpG13	1.85	1.81	3.13	<0.001*	1.50	1.43	1.61	0.006*
FKBP5								
Overall	87.69	86.35	87.78	0.224	4.47	4.46	4.47	0.176
CpG1	83.48	83.74	84.02	0.572	4.43	4.42	4.43	0.849
CpG2	91.90	88.95	91.53	0.056	4.52	4.48	4.52	0.072

*Note.* *p < 0.05, Adjusted models show log-transformed means and group comparisons adjusted for gravida and multiple comparisons.

### 3.2. Association between trauma groups and placental NR3C1 and FKBP5 methylation

**Figure 1.**
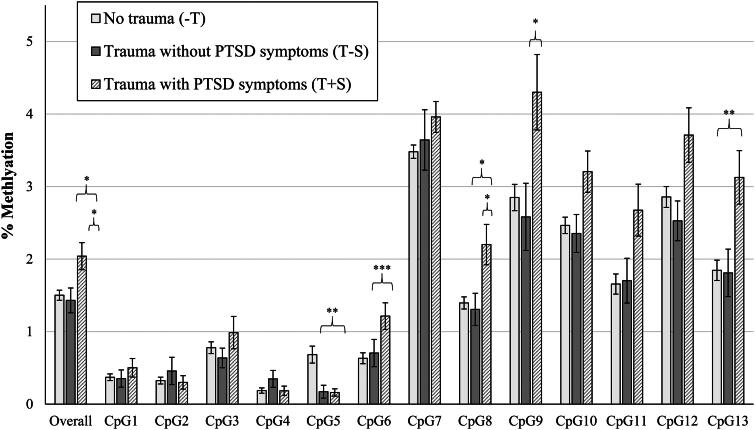
Mean (SEM) of % *NR3C1* Methylation, overall and at each CpG site. Note. *p < 0.05; **p < 0.01; ***p < 0.005. Raw mean (SEM) scores are depicted here prior to logarithmic transformations. Significant between group differences are depicted based on pairwise comparisons controlling for gravida and adjusted for multiple comparisons with Bonferroni correction.

**Figure 2.**
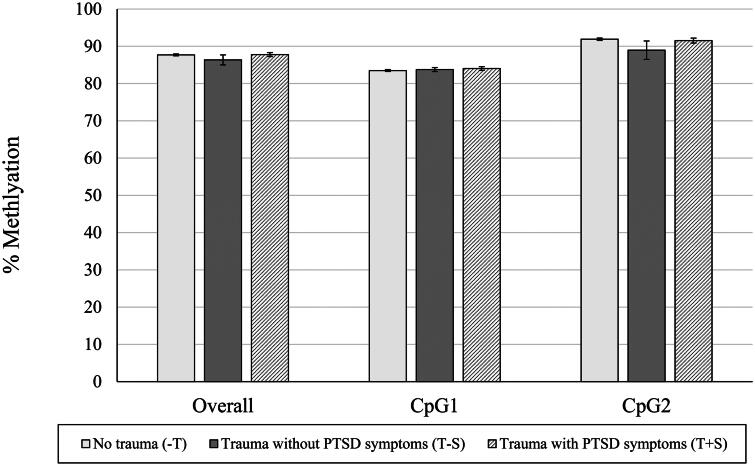
Mean (SEM) of % *FKBP5* Methylation, overall and at each CpG site. *Note.* *p < 0.05, **p < 0.01, ***p < 0.005. Raw mean (SEM) scores are depicted here prior to logarithmic transformations. Significant between group differences are depicted based on pairwise comparisons controlling for gravida and adjusted for multiple comparisons with Bonferroni correction.

Figure 1 shows differences between groups in raw means for percentage of *NR3C1* methylation (*n* = 166 with available data on *NR3C1*) overall and at each individual CpG site. There was a statistically significant effect of trauma group categorization on percentage of placental gene methylation across all CpG sites (*F*(2,165) = 4.797, *p* = 0.009), where the T + S group had significantly higher percentages of methylation overall compared to the −T and T − S groups (T + S vs. −T, *p* = 0.020; T + S *vs.* T − S, *p* = 0.009). For individual CpG sites, there was a statistically significant effect of trauma group categorization on the percentages of placental gene methylation at the CpG5 (*F*(2,165) = 5.985, *p* = 0.003), CpG6 (*F*(2,165) = 5.611, *p* = 0.004), CpG8 (*F*(2,165) = 4.650, *p* = 0.011), CpG9 (*F*(2,165) = 4.517, *p* = 0.012), and CpG13 sites (*F*(2,165) = 5.309, *p* = 0.006). Specifically, compared to the − T group, the T + S group had a significantly lower percentage of methylation at the CpG5 site (*p* = 0.009), but significantly higher percentages of methylation at the CpG6 (*p* = 0.003), CpG8 (*p* = 0.021), CpG9 (*p* = 0.019), and CpG13 (*p* = 0.006) sites. The T + S group also had a significantly higher percentage of methylation at the CpG8 site compared to the T − S group (*p* = 0.037), but there was not a statistically significant effect of trauma group categorization at the CpG7 (*F*(2,165) = 1.326, *p* = 0.268) or CpG4 (*F*(2,165) = 2.259, *p* = 0.108) sites. In examining percentage of *FKBP5* methylation at each site (*n* = 156 with available data on *FKBP5*), there were no significant effects of trauma group categorization on placental gene methylation overall, or at either the CpG1 or CpG2 site (*ps* > 0.05), as shown in Figure 2. See Table 2 for unadjusted and adjusted group comparisons of placental *NR3C1* and *FKBP5* methylation.

## 4. Discussion

This study provides suggestive evidence that prenatal PTSD symptoms *uniquely* contribute to patterns of placental *NR3C1* methylation that are not solely explained by trauma exposure alone. Overall, we found the Trauma + PTSD Symptoms (T + S) group demonstrated higher placental *NR3C1* methylation compared to the No Trauma (−T) and Trauma without PTSD symptoms (T − S) groups. Specifically, the T + S group had higher methylation at the CpG6, CpG8, CpG9, and CpG13 sites compared to the −T group, with the exception of the CpG5 site, as well as higher methylation at the CpG8 site compared to the T − S group.

Although this study is the first, to our knowledge, to investigate the impact of both trauma exposure and PTSD symptoms on placental *NR3C1* methylation, findings complement prior studies of maternal stress, broadly defined. A meta-analysis of seven studies concluded that different types of prenatal stress are associated with increased offspring CpG site-specific methylation in the exon 1 F of *NR3C1* (Palma-Gudiel, Córdova-Palomera, Eixarch, et al., 2015). However, whether higher placental *NR3C1* methylation associated with maternal PTSD symptoms impacts infant development remains to be investigated. Prior studies suggest greater methylation of placental genes may selectively affect some infant outcomes but not others, such as infant temperament (Finik et al., 2020). Greater placental methylation related to maternal psychopathology, in particular, may confer risk for poorer infant development (Conradt et al., 2013). Compared to infants with mothers who did not report depression, infants of depressed mothers with greater placental *NR3C1* CpG2 methylation demonstrated poorer self-regulation, more hypotonia, and more lethargy on neurobehavioral assessments (Conradt et al., 2013). Future research is needed to elucidate whether and precisely which epigenetic mechanisms mediate the effects of maternal trauma exposure and PTSD symptoms on infant stress response and neurobehavioral development.

Our findings highlight the importance of identifying factors that may offset the impact of maternal stress on the infant stress response and development. Animal studies with mice have shown that a stress reduction intervention (i.e. chew toy) buffered the effects of prenatal stress on *NR3C1* methylation (Kubo et al., 2019). In addition to reducing maternal stress, reducing infant stress reactivity via the buffering effects of maternal caregiving on *NR3C1* methylation may be another point of intervention (Conradt et al., 2019). Interestingly, this buffering effect was only observed in female infants, indicating the effect of maternal care behaviors may vary by infant sex.

In contrast to our findings for *NR3C1*, our exploratory analyses of *FKBP5* did not show significant differences in placental gene methylation at either CpG1 or CpG2 site based on trauma group categorization. Although *NR3C1* and *FKBP5* work in concert to regulate the glucocorticoid response pathway, prior factor analytic work on the association between placental methylation and infant neurodevelopment profiles has shown that, while many of the *NR3C1* CpGs loaded onto the same methylation factor, *FKBP5* CpG1 and CpG2 did not load onto any factor (Paquette et al., 2015), and so it is not surprising to only identify relationships with one of these genes. In contrast to studies of Holocaust survivors and their offspring (Bierer et al., 2020; Yehuda et al., 2016), we did not find evidence of transmission of maternal trauma via *FKBP5* methylation, which may be due to differences in trauma type, population, or samples (blood *vs.* placental tissue). The lack of significant associations of trauma exposure or PTSD symptoms with placental *FKBP5* methylation in this study can be considered in the context of a study that examined the association between warzone trauma and placental *FKBP5* methylation (Kertes et al., 2016). The effect of warzone trauma was only observed at one site within the gene body and no effect of warzone trauma was observed within the gene promoter region. The differential effects of trauma exposure by placental *FKBP5* gene region may be attributable to different trauma types across studies and highlight further work is needed to reconcile differential findings across studies.

Our results also showed that trauma-exposed individuals who reported PTSD symptoms during pregnancy exhibited significantly different patterns of placental *NR3C1* methylation than trauma-exposed individuals who did not report PTSD symptoms. Although further research and replication is needed in order to explore mechanisms and other pathways of trauma transmission, our findings highlight that interventions to support maternal mental health and wellbeing for trauma survivors may disrupt one potential pathway of transmission of maternal trauma. Pregnancy and childbirth can be especially challenging for survivors of trauma due to pregnancy-related body changes and sensations, increased risk of pregnancy complications, a heightened sense of vulnerability, and invasive medical procedures (Gokhale et al., 2020). The American College of Obstetricians and Gynecologists (ACOG) recommends screening for trauma in each trimester and postpartum, including lifetime sexual violence (ACOG, 2019). With effective screening, a history of trauma exposure can be identified and resources can be provided to reduce the likelihood of development of PTSD and promote resilience, including behavioral health referrals, doula support, and trauma-focused patient education (Rosenblum et al., 2017). Since screening for trauma exposure and related symptoms can be difficult in a busy clinical setting and not all survivors may choose to disclose to providers, a universal trauma-informed approach to care can reduce the likelihood of inadvertently re-traumatizing patients during routine care procedures and decrease patients’ susceptibility to develop PTSD symptoms during pregnancy (Ward, 2020). Overall, it remains important to promote and emphasize the mental health of women during pregnancy to mitigate the potential maternal-fetal transmission of stress and trauma.

Results should be considered in the context of limitations. Although our sample was racially/ethnically diverse and included predominantly low-income women, generalizability to national and international or non-English speaking populations may be limited due to the cross-sectional design examining English-speaking, pregnant women in one Northeast U.S. region. As small effect sizes were shown in this proof-of-concept pilot study, actual effect sizes may also be larger than observed in this study. Additionally, the use of a gold-standard assessment, such as the Clinician-Administered PTSD Scale, may allow for future investigation of whether dose-response relationships exist between PTSD symptom severity and degree of placental gene methylation. Use of the candidate gene approach is another limitation of this study and future studies should employ genome-wide approaches and other biological markers of stress (e.g. cortisol) to studying complex biobehavioral processes underlying transmission of trauma. In particular, comparisons of methylation patterns in placental samples *vs*. maternal whole blood may elucidate epigenetic differences at the maternal-fetal interface. We also recognize that the placenta is a heterogeneous tissue, and although we performed sampling and sample preparation steps to reduce heterogeneity, there can be differences in the underlying cellular populations within samples that could be confounding our results. Finally, given consistent sex differences in clinical and preclinical prenatal programming studies and in glucocorticoid signaling, future research should examine sex differences and/or stratify by infant sex to investigate the impact of PTSD symptoms on placental methylation levels (Bulka et al., 2023).

## 5. Conclusion

This proof-of-concept study is the first to demonstrate the impact of prenatal PTSD symptoms on epigenetic regulation of placenta glucocorticoid signaling. Specifically, trauma survivors with prenatal PTSD symptoms exhibited differential patterns of methylation for multiple placental *NR3C1* sites compared to women without trauma exposure or PTSD symptoms. Future research is needed to investigate mechanisms of this association, impact on infant neurodevelopment, and genome-wide approaches. Results highlight the critical importance of effective interventions to support perinatal mental health for survivors of trauma.

## Supplementary Material

Supplemental Material

## CRediT author statement

Laura R. Stroud: Conceptualization, Methodology, Investigation, Resources, Supervision, Project Administration, Funding Acquisition, Writing – Review and Editing.

Nancy C. Jao: Conceptualization, Methodology, Formal Analysis, Data Curation, Visualization.

L. G. Ward: Writing – Original Draft.

Sharon Y. Lee**:** Writing – Original Draft.

Carmen J. Marsit: Supervision, Writing – Review and Editing.
