# Supplementary material for: Differential impact of prenatal PTSD symptoms and preconception trauma exposure on placental NR3C1 and FKBP5 methylation
Source: Stress. 2024 Apr 26;27(1):2321595. doi: 10.1080/10253890.2024.2321595 (PMC11238900; doi:10.1080/10253890.2024.2321595)
Supplement: Supplemental Material [file ISTS_A_2321595_SM2740.docx]

**Supplementary Table 1.** *Enrollment Flow Chart*

**Not Enrolled (*n*=436)**

Excluded during screening process

## Available FKBP5 data

## (*n*=156)

## Available NR3C1 data

## (*n*=166)

**Excluded from analyses (*n*=7)**

Late miscarriage (*n*=1)

Enrolled in other study (*n*=4)

Baseline data only (*n*=2)

## Enrolled in Parent Study (*n*=205)

**Assessed for Eligibility via Phone Screen (*n*=641)**

**Supplementary Table 2.** *Genomic Coordinates*

| **Gene and CpG Site** | **Genomic Coordinates** |
| --- | --- |
| **NR3C1**  CpG1  CpG2  CpG3  CpG4  CpG5  CpG6  CpG7  CpG8  CpG9  CpG10  CpG11  CpG12  CpG13 | Chr 5: 142783592  Chr 5: 142783599  Chr 5: 142783602  Chr 5: 142783608  Chr 5: 142783611  Chr 5: 142783501  Chr 5: 142783503  Chr 5: 142783513  Chr 5: 142783519  Chr 5: 142783533  Chr 5: 142783555  Chr 5: 142783570  Chr 5: 142783573 |
| **FKBP5**  CpG1  CpG2 | Chr 6: 35558488  Chr 6: 35558514 |
